# Binding of Kif23-iso1/CHO1 to 14-3-3 Is Regulated by Sequential Phosphorylations at Two LATS Kinase Consensus Sites

**DOI:** 10.1371/journal.pone.0117857

**Published:** 2015-02-06

**Authors:** Didier Fesquet, Geoffroy De Bettignies, Michel Bellis, Julien Espeut, Alain Devault

**Affiliations:** CRBM UMR 5237, CNRS and Université de Montpellier, Montpellier, France; University of Padova, ITALY

## Abstract

Kif23 kinesin is an essential actor of cytokinesis in animals. It exists as two major isoforms, known as MKLP1 and CHO1, the longest of which, CHO1, contains two HXRXXS/T NDR/LATS kinase consensus sites. We demonstrate that these two sites are readily phosphorylated by NDR and LATS kinases *in vitro*, and this requires the presence of an upstream -5 histidine residue. We further show that these sites are phosphorylated *in vivo* and provide evidence revealing that LATS1,2 participate in the phosphorylation of the most C-terminal S814 site, present on both isoforms. This S814 phosphosite was previously reported to constitute a 14-3-3 binding site, which plays a role in Kif23 clustering during cytokinesis. Surprisingly, we found that phosphorylation of the upstream S716 NDR/LATS consensus site, present only in the longest Kif23 isoform, is required for efficient phosphorylation at S814, thus revealing sequential phosphorylation at these two sites, and differential regulation of Kif23-14-3-3 interaction for the two Kif23 isoforms. Finally, we provide evidence that Kif23 is largely unphosphorylated on S814 in post-abscission midbodies, making this Kif23 post-translational modification a potential marker to probe these structures.

## Introduction

NDR/LATS kinases form a specific subgroup in the AGC kinase family and are present throughout the eukaryotic domain, including protists. NDR/LATS are characterized by their activation through binding to MOB proteins and phosphorylation by a member of the MST or YSK subgroups of the STE20 kinase family. The NDR/LATS clade itself comprises two distinct members which are called NDR and LATS in animals. These two subgroups are duplicated as NDR1,2 and LATS1,2 in vertebrates.

NDR/LATS kinases participate in a wide variety of cellular processes including mitotic exit, polarized cell growth and control of cell proliferation [1]. Most functional investigations in animals have focused on LATS as a core component of the hippo pathway [2,3]. This pathway is involved in inhibition of cell proliferation in high cell density environments or structured epithelia, as well as mecano-transduced differentiation processes [4]. On the other hand, few studies have unraveled potential roles for NDR/LATS kinases in mitosis. NDR has been proposed to promote G1/S transition [5] and to control centrosome duplication [6] and chromosome alignment [7], while LATS was found necessary for proficient cytokinesis [8,9]. Actually, better insight on the mitotic functions of NDR/LATS was gained in yeast. Dbf2/Dbf20 of *S. cerevisiae* and Sid2 in *S. pombe* are key players respectively of the MEN (Mitotic Exit Network) and SIN (Septum Initiation Network) pathways, whose activations are essential for cytokinesis [10], [11]. In both organisms, these kinases directly phosphorylate and activate Cdc14/Clp1 phosphatase [12][13], and Cdc14 in *S. cerevisiae* is necessary to remove phosphorylations on Cdk substrates and enter cytokinesis. Other Dbf2 substrates more directly involved in cell cleavage include Chs2 [14]) and Hof1 [15]. However, transposing these findings in animals is not straightforward, not to mention differences in cytokinetic processes between yeasts and animals. First, Dbf2 and Sid2 are more distantly related to animal NDR/LATS kinases than is Cbk1 and Orb6, the other NDR/LATS yeast kinases, and which are involved in polarized cell growth [16], [17]. Second, all the Dbf2 and Sid2 phosphorylated sites identified so far have a minimal AGC kinase RXXS/T signature, which matches that found using degenerate peptide libraries for Dbf2 [18]. On the other hand, a more specific HXRXXS/T consensus has emerged for both animal LATS/NDR and yeast Cbk1 kinases [19], [20]. A strong requirement for the histidine in position-5 of the phosphorylated serine or threonine was found for these kinases, using either degenerate peptides or specific physiological protein substrates [19][21]. This requirement is unique among all AGC kinases subgroups, which can otherwise often phosphorylate the same substrates [19,22]. It is worth noting, however, that not all NDR/LATS identified substrates bear this-5 histidine [1].

In the present work, we have focused on kinesin Kif23 as a candidate target of NDR/LATS kinases, as it contains consensus HXRXXS/T phosphorylation sites. Kif23 is a member of the kinesin-6 subgroup and forms a heterotetramer complex with MgcRacGAP. This complex, also called centralspindlin, is involved in stabilization of the mitotic spindle anti-parallel microtubules, from anaphase onset to abscission [23]. In the absence of Kif23 or MgcRacGAP, cells undergo furrow constriction but fail to complete cytokinesis. Binding of centralspindlin to the mitotic spindle is regulated by phosphorylation of Kif23 in two ways. First, Cdk1 phosphorylation of the N-terminal motor domain inhibits microtubule binding until the metaphase to anaphase transition, when Cdk1 activity drops [24]. Then, Aurora B, relocalised at the spindle midzone at anaphase, phosphorylates Kif23 at S708/S812 (numbering according to the two major isoforms), which allows its stable binding to midzone microtubules [25]. Actually, S708/S812 phosphorylation does not increase directly Kif23 interaction with microtubules. It rather counteracts the sequestration of Kif23 by 14–3–3. In fact, Kif23 binds to 14–3–3, due to the constitutive phosphorylation of S710/S814 on Kif23 by an as yet unidentified kinase. This 14–3–3 phospho-binding site is disrupted when S708/S812 becomes phosphorylated by Aurora B. Consequently, replacement of wild type Kif23 by Kif23 S708A mutant leads to severe cytokinetic defects. On the other hand, S710A Kif23 mutant shows no defect in cytokinesis, but induces aberrant clustering of Kif23 due to disruption of its binding to 14–3–3 [25]. In this study, we provide evidence that LATS1,2 are involved in generating this 14–3–3 binding site by directly phosphorylating Kif23 on S710/S814 *in vivo*. We also identify a new *in vivo* phosphorylation site (pS716) present specifically on the longer isoform, and which is necessary for phosphorylation at S814 and 14–3–3 binding. Finally, we show that dephosphorylation of pS710/pS814 is a post-abscission process that is uncoupled from Kif23 degradation.

## MATERIALS AND METHODS

### Antibody reagents

Affinity purified anti-Kif23 was prepared from rabbit serum immune to human GST-Kif23-iso1(682–907). Antibodies directed against pS716 (ab pS716) and pS814 (ab pS814) of Kif23-iso1 were prepared as follows. Rabbits were immunized against peptides RRSNpSCSSISVAC and RRSRpSAGDRWVDC, where pS denotes phospho-serine and C at the C-terminus was used for crosslinking to thyroblobulin. Rabbits were handled in the animal house of the Institut Universitaire de Technologie de Montpellier which has an institutional agreement (number D34–172–8) from Direction Départementale de la Protection des Populations (DDPP) de l’Hérault (Montpellier, France), which operates under the supervision of the Ministry of Agriculture and is dedicated to rabbit immunization and blood sampling. The protocol was not further submitted to the approval of an ethics committee. Such approval was not necessary for those experiments under the French and European legislation at the time they were conducted. At the end of the immunization protocol, rabbits were anaesthetized with pentobarbital (30mg/kg) and then sacrificed by injection of dolethal (pentobarbital, 220 mg/kg). Rabbit sera were first purified on the peptides above immobilized as BSA conjugates on CNBr-activated sepharose, and the eluates where rapidly passed over unphosphorylated peptide-coupled resin. Anti-myc antibodies where prepared from mouse 9E10 hybridoma. Anti-human LATS1 clone C66B5 was from Cell Signalling Technology, anti-human MgcRacGAP from abcam (ab2270), anti-human cyclin B1 from Santa Cruz (sc-752), anti-Flag clone M2 and anti-tubulin clone DMA1 from Sigma.

### Plasmids

For expression of 6his fusion proteins in *E. coli*, fragments of human Kif23-iso1 (a.a. 645–911, NM_138555.2), human PARD3 (a.a. 2–157, BC071566.1), human CYLD (a.a. 2–150, BC012342.1), human TSG101 (a.a. 240–390, BC002487.1) and mouse MTSS1 (a.a. 293–443, BC024131.1) were cloned by PCR C-ter of 6xhis tag in pRSETA. Kif23-iso1 (a.a. 682–907, NM_138555.2) was also cloned in pGEX-4T1 for expression of GST-Kif23. For expression of Nter-tagged fusion proteins in human cells, complete open reading frames of human Kif23-iso1 (BC071566.1), *X. laevis* NDR1 (NM_001086949.1) and X. *laevis* MOB1A (NM_001089248.1) were cloned in pCMV10–3xFlag, pRK5HAGST and pRK5myc, respectively. Human 14–3–3, Kif23iso1 and LATS2 were also expressed from vectors pEF6-myc-14–3–3γ [26], pEGFPC1-KIF23-iso1 [27], pCMV-myc-LATS2 [28] and pEGFP3B-LATS2 [28], all as Nter-tagged fusion proteins. To express kinase-dead mutants, we generated pRK5HAGST-NDR1-K118A by site-directed mutagenesis, while pEGFP3B-LATS2–K697M was kindly provided by H. Nojima [28].

### Recombinant proteins

Recombinant proteins were produced in BL21 pLysS *E. coli* strain and purified on either TALON affinity resin (6xhis-tagged proteins) (Clontech) or glutathione-sepharose (GST-tagged proteins).

### Cell culture

HeLa and HEK293T cells were cultured in DMEM with fetal calf serum. HEK293T were transfected with polyethylenimine. For double thymidine block synchronization, HeLa cells were incubated in 2.5 mM thymidine for 24 hrs, washed and released for 8 hrs and incubated again in 2.5 mM thymidine for 16 hrs. Synchronised cells were then released in normal medium and sampled at different time points. siRNA experiments were carried out using RNAiMAX (Invitrogen). Sequence GCAGUCUUCCAGGUCAUCU of Kif23 (iso1 and 2) [29] was used as target for RNAi. LATS 1 and 2 were depleted using either one of two sets of siRNAs: ACUUUGCCGAGGACCCGAA (LATS1) and GGACCAAACAGUGACACUU (LATS2), set 1 [30], and CACGGCAAGAUAGCAUGGA (LATS1) [4] and AGCAGAUUCAGACCUCUCC (LATS2) [31], set2. HEK293T cells were transiently transfected using JetPEI and collected 40 hrs after transfection. Cell extracts were prepared in 20 mM Tris pH8.0, 150 mM NaCl, 1% IGEPAL, 5mM EDTA, 50 mM NaF, 50 mM β-glycerophosphate, 1 mM DTT, 1mM PMSF, 1 mM sodium vanadate and complete protease inhibitor tablet (Roche).

### NDR1-MOB1A and LATS2–MOB1A kinase preparation

HEK293T cells were transfected with pRK5HAGST-NDR1/pRK5-Myc-MOB1A or pEGFP3B-LATS2/pRK5-Myc MOB1A. 40 hrs after transfection, cells were treated for 1 hour with 1 μM okadaic acid. NDR1 and LATS2 kinases were adsorbed to glutathione beads (via a GST-tagged GFP trap kindly provided by A. Lamond (Dundee) for LATS2) and eluted with glutathione. Kinase-dead versions were similarly prepared. The GFP-trap used consists of a camel anti-GFP antibody fused with GST. Kinase preparations were analyzed by gel analysis (S1 Fig.).

### Immunofluorescense and microscopy

Cells were fixed in 4% paraformaldehyde, 0.5% Triton X-100, 1X BRB80 for 20 minutes and processed for immunofluorescence staining with all antibodies diluted in PBS-1% BSA buffer. To obtain Kif23 and pS814 staining on the same cells, these where first stained with ab pS814 and Alexa 488-coupled anti-rabbit, and then stained with anti-Kif23 coupled to Cy3 fluorophore. Z-stacks were acquired and processed into maximum intensity projections. Midbody signals were quantified using ImageJ (http://rsb.info.nih.gov/ij/).

### Real-time qRT-PCR

Quantification of LATS2 mRNA by qRT-PCR was performed using oligos GGGTTCAGGTGGACTCACAA and GTCCCCACACCGACAGTTAG. GAPDH was used as the reference gene.

### Statistical analysis

For statistical analysis of quantified data from Western blots, a one-sample two-tailed t-test was used, while for quantified data from immunofluorescence microscopy, we used a two-tailed Mann-Whitney test.

## RESULTS

### Identifying Kif23 S716 and S814 as *in vitro* targets of NDR and LATS kinases

**Fig 1:**
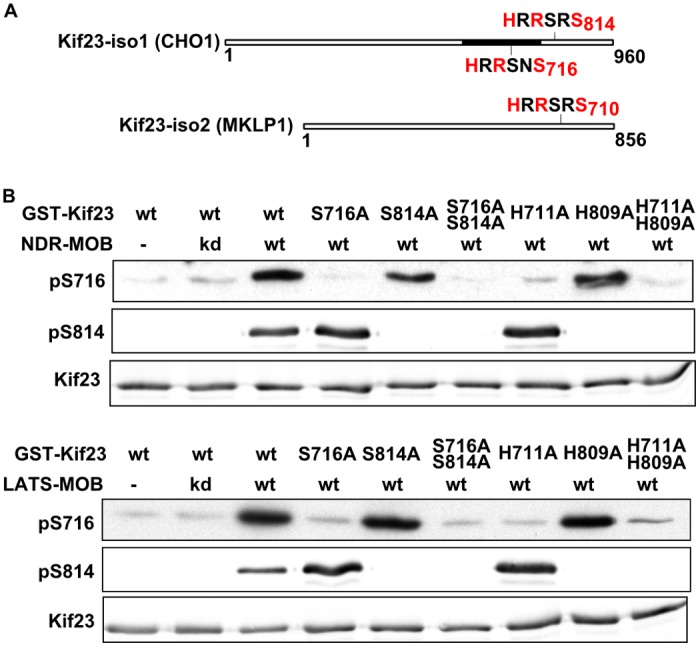
*In vitro* phosphorylation of Kif23 on S716 and S814 by NDR1-MOB1A and LATS1–MOB1A. A. Schematic representation of Kif23 isoforms. Black box in Kif23-iso1 depicts the 104 amino acids insertion corresponding to exon 18. B. WT and mutant GST-tagged Kif23-iso1 (a.a. 682–907) were incubated with active NDR1-MOB1A or LATS2–MOB1A and analysed by SDS-PAGE and western blotting with the indicated antibody.

With the aim of identifying new mitotic substrates for NDR/LATS kinases, we performed a simple bioinformatics screen. A scansite search was run to extract a list of HxRxxS/T motif containing proteins from the UniProtKB/Swiss-Prot database. This list was further filtered with the “cell cycle” Gene Ontology term and for the presence of evolutionary conservation in animals ranging from drosophila to humans. From a primary list of nearly 50 candidates, 9 were chosen for their acknowledged role during mitosis and were expressed in *E. coli* as 6-His tagged ~20 kDa domains. Among those, five could be radiolabeled when incubated with ATP-γ-P^33^ and NDR1-MOB1A kinase purified from HEK293T cells (S1 and S2 Figs.). We then determined if substituting alanine for serine in the HXRXXS consensus sequence affected phosphorylation by the NDR kinase. Two candidate substrates indeed showed decreased phosphorylation when mutated: Kif23 and PARD3 (S2 Fig.). Interestingly, the identified phosphosites for Kif23 and PARD3 were documented in large scale phospho-proteomics analysis (S1 Table) [32,33]. We chose to concentrate on Kif23 kinesin for its well established essential role during cytokinesis. Kif23 exists as two isoforms in mammals, arising from differentially spliced transcripts: isoform 1 (also known as CHO1) and isoform 2 (also known as MKLP1), the later lacking a 104 amino acid domain near the C-terminal end (Fig. 1A). Isoform 1 possesses two candidate NDR/LATS sites, HRRSNS716 and HRRSRS814, the former located in the supplementary domain, and which is therefore absent in isoform 2. We will thereafter refer to the Kif23 isoform1 (Kif23-iso1) sequence for numbering the position of these phosphosites. Two polyclonal antibodies, ab pS716 and ab pS814, were raised against these two phosphosites and their specificity verified by a dot blot assay (S3 Fig.). These allowed better monitoring of Kif23 phosphorylation by NDR/LATS kinases. Recombinant GST-Kif23-iso1(682–911) which includes most of the tail domain of Kif23 was used as a substrate. Fig. 1B shows that both phosphosite antibodies detected wild type Kif23-iso1(682–911) after incubation with either NDR1 or LATS1 kinases. These phospho-specific signals were reduced to control levels when the corresponding serines were mutated to alanines. We then asked if the histidines at position-5 were important for efficient phosphorylation, as has been shown for other known LATS substrates [19][21]. As shown in Fig. 1B, H711A and H809A Kif23 mutants exhibited only background signals after treatment with NDR and LATS kinases when detected with ab pS716 and ab pS814, respectively, revealing a strong dependency on these residues for Kif23 phosphorylation at both sites. We conclude that *in vitro*, S716 and S814 of Kif23 are specific phosphorylation sites for NDR1 and LATS1 kinases.

### *In vivo* phosphorylation of Kif23 on S716 and S814

**Fig 2:**
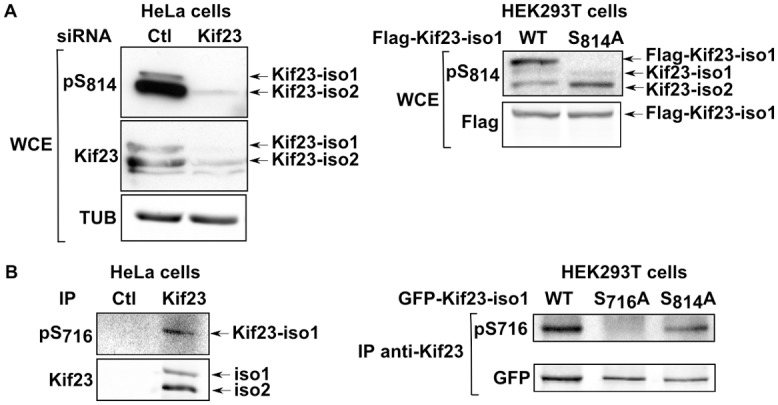
*In vivo* phosphorylation of S716 and S814 on endogenous and exogenous Kif23. A. Detection of S814 phosphorylation by Western blot on whole cell extracts (WCE) of HeLa cells treated or not with Kif23 siRNA (left) or WCE of HEK293T cells transfected with WT and mutated Flag-tagged Kif23-iso1 (right). B. Detection of S716 phosphorylation on material immunoprecipitated with anti-Kif23 antibody from HeLa cells (left) or HEK293T cells transfected with WT and mutated GFP-tagged Kif23-iso1 (right).

While phosphorylations at S716 (isoform 1) and S814 (isoforms 1 and 2) were both reported in phospho-proteomic studies (S1 Table), only the latter has been characterized in more detail [25]. We could confirm with our anti-pS814 antibody that Kif23 is phosphorylated *in cells* on S814 for both endogenous isoforms in HeLa cells (Fig. 2A, right panel) as well as on exogenous Kif23-iso1 expressed in HEK293T cells (Fig. 2A, right panel). Albeit of lower quality, our antibody directed against pS716 could detect endogenous and ectopically expressed pS716–Kif23-iso1 on anti-Kif23 immunoprecipitates (Fig. 2B), validating the existence of this phospho-residue on endogenous Kif23. However, ab pS716 did not permit us to conduct intracellular localisation studies by immunofluorescence. On the other hand, ab pS814 strongly labeled the central spindle and midbody rings from anaphase initiation to late telophase (S4 Fig.) in fixed HeLa cells, as previously described with another pS814 antibody [25]. To see if S814 phosphorylation levels varied during mitosis, we performed Western blot analysis on extracts of HeLa cells released from a double thymidine block. As shown in S5 Fig., levels of pS814 mirrored those of Kif23 for both isoforms from late G1 to exit of mitosis, suggesting that phosphorylation of this site is constitutive. We conclude that Kif23 is phosphorylated *in cells* on LATS/NDR consensus sites S716 and S814 for both isoforms, with the latter being detected in anaphase, telophase, and cytokinesis.

### LATS is involved in phosphorylation of Kif23 on S814 *in vivo*

**Fig 3:**
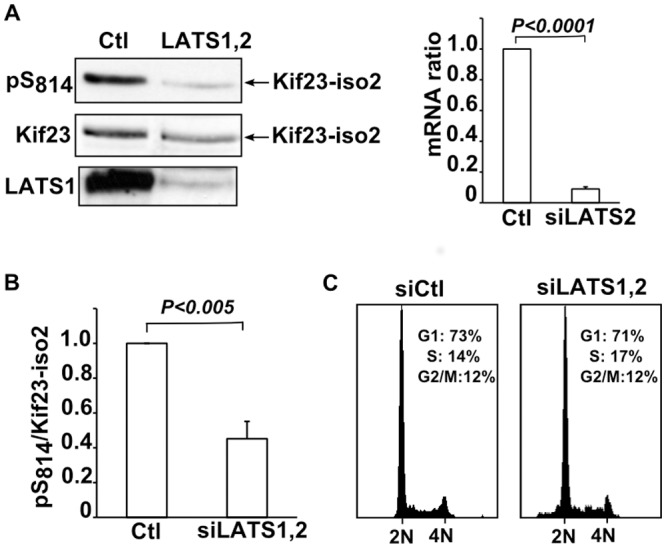
pS814–Kif23 levels are reduced after depletion of LATS1,2. A. Unsynchronised HeLa cells were treated with LATS1,2 siRNAs (set1, see Materials and methods) for 72 hrs and analysed for pS814–Kif23 levels. Depletion of Lats1 was monitored by Western blot and that of LATS2 by RT-PCR (right panel). Ability of LATS2 siRNA (set 1) to deplete LATS2 protein was verified indirectly on exogenous myc-LATS2 (S6 Fig.). B. Quantification of pS814 levels corrected for the amounts of Kif23 (pS814/Kif23) from A and three other similar experiments.

**Fig 4:**
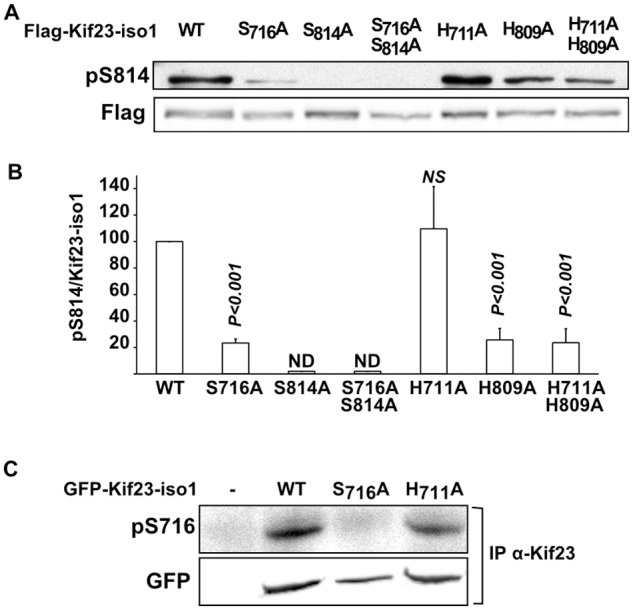
Analysis of pS716 and pS814 levels on WT and mutant Kif23 in HEK293T cells. A. WT and mutant Flag-tagged Kif23-iso1 were expressed in HEK293T cells and analysed for S814 phosphorylation by Western blot. B. Histograms of pS814/Kif23 ratios calculated from A and three other experiments. ND: not detected; NS: not significant. P values refer to comparisons between WT and specified mutant Kif23. C. WT and mutant GFP-tagged Kif23-iso1 were expressed in HEK293T cells, immunoprecipitated with anti-Kif23 antibody and analysed for S716 phosphorylation.

The kinase responsible for *in vivo* phosphorylation of S814 has not been identified yet. We focused on testing whether LATS kinases regulate Kif23 phosphorylation in human cells, and did not address NDR kinases in our cellular settings. LATS1 and 2 were co-depleted using two different pairs of previously validated siRNAs [4,30,31]. Results obtained with set1 siRNAs are presented in Fig. 3. While LATS1 and 2 were strongly depleted, pS814 to Kif23 ratios were more modestly reduced by 56% (Fig. 3A,B). The absence of a more complete reduction of pS814 levels might be explained by redundancy of NDR/LATS kinases, incomplete LATS1,2 depletion, differential stabilities of the phosphorylated and unphosphorylated forms of Kif23, or involvement of other kinases. Cells depleted of LATS1 and 2 did not exhibit an altered FACS profile for DNA content (Fig. 3C), excluding the possibility that the observed changes in Kif23 S814 phosphorylation levels could result from enrichment in a particular phase of the cell cycle. To strengthen our conclusion that LATS1 and 2 were involved in S814 phosphorylation, we wished to determine if the LATS consensus histidine upstream S814 was important for the observed phosphorylation level of this site *in vivo*. Wild type and mutated Flag-tagged Kif23-iso1 were expressed in HEK293T cells and analyzed for their levels of pS814. Fig. 4A and B shows that mutating the upstream histidine, which was shown to be necessary for S814 phosphorylation by LATS *in vitro*, decreased *in vivo* phosphorylation 4 fold. Given that NDR/LATS kinases are the only known basophilic kinases showing specificity for histidine at position-5, together with the siRNA results, we conclude that LATS1 and 2 significantly participates in Kif23 phosphorylation at S814 *in vivo*. On the other hand, the presence of a histidine at position-5 of S716 was dispensable for its phosphorylation, arguing that NDR/LATS is not involved in this phosphorylation (Fig. 4C).

### Phosphorylation of S814 is dependent on S716 phosphorylation on Kif23-iso1

**Fig 5:**
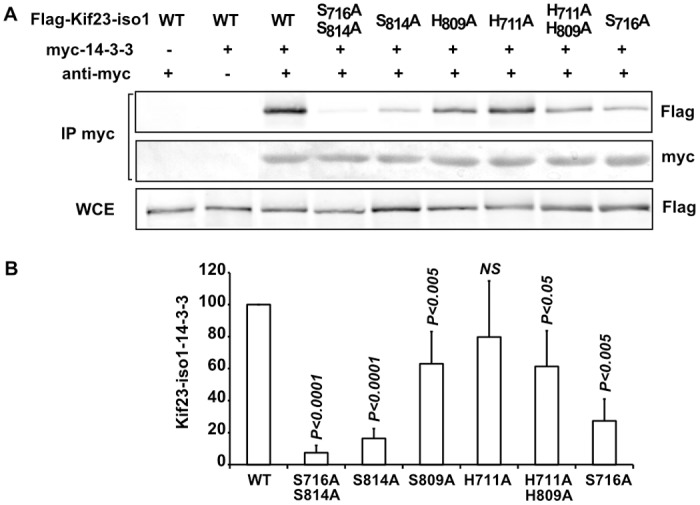
Phosphorylation dependant interaction between Kif23 and 14–3–3. A. WT and mutant Flag-tagged Kif23-iso1 were expressed with myc-tagged 14–3–3 in HEK293T cells. Material immunoprecipitated with anti-myc antibodies was analysed by Western blot for the presence of Kif23. B. Histograms show amounts of Flag-Kif23 present on anti-myc immunoprecipitates corrected by the amount of Flag-Kif23 in extracts, calculated from A and three other experiments. P values refer to comparisons between WT and specified mutant Kif23.

Unexpectedly, mutation of S716 to alanine not only impeded phosphorylation at S716 (Fig. 2B), it also strongly reduced that of S814 on isoform 1 (Fig. 4). On the opposite, mutation of S814 in Kif23-iso1 did not affect phosphorylation of S716 (Fig. 2B). Meanwhile, the shorter Kif23-iso2, lacking the S716 phosphosite, exhibited similar levels of S814 phosphorylation to that of Kif23-iso1 (S7A Fig.), comforting the conclusion that phosphorylation of S716 is necessary for phosphorylation on S814, rather than allowing hyper-phosphorylation of S814. This suggests that an ordered sequence of phosphorylation is taking place, with that on S716 occurring before S814. This could be due to unphosphorylated domain around S716 interacting with and masking S814. It was previously demonstrated that Kif23-iso2 interacts with 14–3–3 in a phospho-S814 dependent manner and that the peptide sequence surrounding phospho-S814 has 14–3–3 binding activity [25]. We could confirm this interaction by immunoprecipitation using tagged versions of Kif23-iso1 and 14–3–3-γ expressed in HEK293T cells (Fig. 5 and S7A Fig.), as well as with both endogenous Kif23 isoforms (S7B Fig.). Mutation of S814 to alanine led, as expected, to a strong, 6 fold decrease in 14–3–3 binding (Fig. 5). Mutation of the upstream H809 but not that of H711, significantly reduced binding to 14–3–3, although more modestly. We then asked if Kif23-iso1 S716A mutant could influence formation of Kif23/14–3–3 complexes. In keeping with its ability to negatively regulate S814 phosphorylation, S716A mutant strongly reduced binding to 14–3–3 (Fig. 5). It ensues that binding of Kif23-iso1 to 14–3–3 depends on the phospho-S814 binding site, which in turn is dependent on prior phosphorylation of the upstream S716. As Kif23-iso1 S814A mutant retained only residual binding capacity to 14–3–3, phospho-S716 is probably not a 14–3–3 binding site per se, and S716D mutation was unable to rescue 14–3–3 binding of the S814 mutant (S8 Fig.). Altogether, these results show that phospho-S814 is the main determinant of 14–3–3/Kif23 complex formation, while phosphorylation of S716 is necessary for that on S814. Since S716 phosphosite is only present in isoform 1, this raises the possibility that the two Kif23 isoforms are differentially regulated.

### Kif23 is hypophosphorylated on S814 in midbody remnants

**Fig 6:**
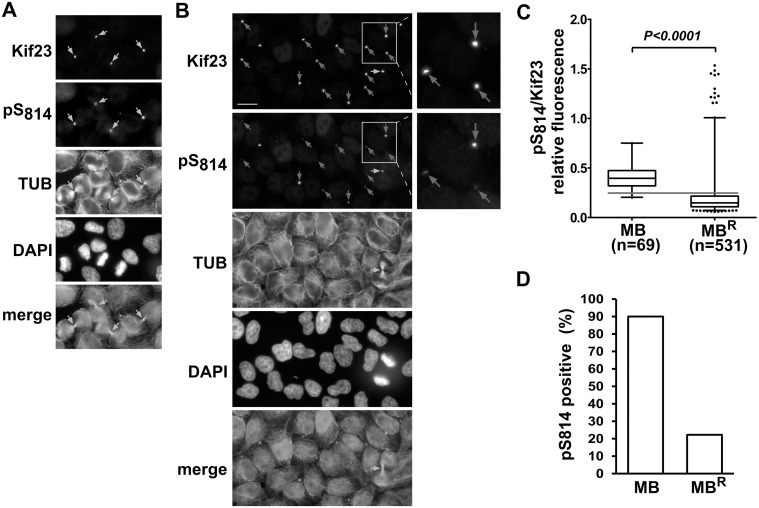
Midbody remnants show low level of Kif23 phosphorylation at S814. A, B. Micrographs of HeLa cells stained with DAPI and antibodies against pS814, Kif23 and tubulin, and representative of cytokinetic MBs (A) or MB^R^s (B). Yellow arrows point to cytokinetic MBs, while red and green arrows point to MB^R^s with low and high pS814 relative staining, respectively. Scale bar: 15 μM. C. Relative phosphorylation at S814 (pS814/Kif23 ratio) was calculated on individual midbodies either engaged in late cytokinesis (MB) or not (MB^R^), and represented as box and whiskers plots. Outliers represent values found outside the 2.5 to 97.5 percentile range. The red horizontal line represents the threshold ratio value above which lie 90% of cytokinetic midbodies. D. Bars represent the percentage of pS814/Kif23 ratio values that are higher than the threshold value shown in C and counted as pS814 positive.

As noticed earlier, we found that midbodies (MB) in cells undergoing cytokinesis are strongly labeled with both anti-Kif23 and anti-pS814 antibodies (Fig. 6A). However, differential labeling with these two antibodies was noticed when examining structures known as midbody remnants (MB^R^), which correspond to post-abscission midbodies. We identified MB^R^s in HeLa cells as Kif23 positive single dotted signals not located in the thin constriction bridge of late cytokinetic cells (Fig. 6B). To strengthen the identity of these structures, we verified that they were also labeled with an anti-MgcRacGAP antibody, as MgcRacGAP is known to persist in MB^R^s (S9 Fig.) [34]. While quite uniformly and strongly labeled by anti-Kif23 antibodies, these MB^R^s produced more heterogeneous signals when revealed by ab pS814, with a large majority exhibiting weak signals (Fig. 6B). To better characterise pS814 levels in these two MB populations, we calculated pS814/Kif23 signal ratios on each individual midbody objects. As shown in Fig. 6C, the median ratio was 2.7 higher for mitotic MBs, as compared to that of MB^R^s, revealing that Kif23 in MB^R^s are much less phosphorylated than in cytokinetic MBs. When we applied a minimal threshold ratio above which 90% of late cytokinetic MBs values were found, only 22% of remnants came out as positive for pS814 (Fig. 6D). This suggests that disappearance of phospho-S814 is not merely a consequence of MB^R^ degradation, but of active dephosphorylation of Kif23 at this site which takes place after abscission and early in the MB^R^ lifespan.

## DISCUSSION

During our search of mitotic substrates for NDR/LATS kinases, we noticed that Kif23 displayed two consensus HXRXXS phosphorylation sites. One of these sites, pS814, was previously shown to act as a 14–3–3 binding site, but the kinase responsible for this phosphorylation was not identified. In this work, we could show that LATS1/MOB1A and NDR1/MOB1A kinases phosphorylate both consensus sites *in vitro*, and that the presence of histidine at position-5 is essential for these phosphorylations. A majority of the identified *in vitro* and in *vivo* NDR/LATS phosphorylated sites comply with the HXRXXS/T signature [1]. Among those, it was verified that phosphorylation of S175 of angiomotin *in vivo* was dependant on the upstream histidine [21]. Studies performed on a degenerate peptide library with yeast Cbk1 or semi-degenerate peptides with LATS1 further showed the strong requirement of the histidine residue [19,20]. When we asked if phosphorylation of S716 and S814 of Kif23-iso1 were also dependant on the presence of a histidine *in vivo*, we obtained different outcomes. While S716 phosphorylation was unaffected by its absence, that of S814 was strongly reduced. As no other basophilic kinase has been reported to rely on a histidine at-5 for substrate recognition, this suggests that S814 is phosphorylated by a NDR/LATS kinase *in vivo*. On the other hand, the neutral outcome of the H711A mutation rather suggests that NDR/LATS are not involved in S716 phosphorylation *in vivo*, in contrast to what we observed *in vitro*. It cannot be excluded though, that NDR/LATS could be less stringent for this site *in vivo*. As compared to mutating the upstream histidine, which resulted in a 4 fold decrease in S814 phosphorylation, a more modest effect emerged when we performed LATS depletion. LATS1,2 depletion resulted in a 56% decrease in S814–phosphorylated Kif23. While it is conceivable that this modest impact may arise from the potentially high redundancy of the four NDR/LATS kinases or incomplete depletion by siRNA, it is also possible that other kinases play a role in this phosphorylation. In that respect, YAP and p21, two *in vivo* NDR/LATS substrates, are also known to be phosphorylated by other kinases [5,35–37]. We conclude from our siRNA results and the strong dependency of S814 on the upstream histidine that LATS contributes to *in vivo* phosphorylation of S814 on both Kif23 isoforms.

While monitoring the phosphorylation status at both S716 and S814 on non-phosphorylatable Kif23-iso1 mutants, we could uncover an interplay between these two sites. Namely, mutation of S716 drastically reduced phosphorylation of the distant S814, but not the opposite. This would imply that phosphorylations at S716 and S814 obey to a hierarchical order in Kif23-iso1, whereby S716 phosphorylation would occur first and be necessary for efficient phosphorylation at S814. We note that this cross-talk between these two phosphorylation events was not observed in our *in vitro* NDR/LATS kinase assay using a ~20 kDa Kif23 fragment. Phosphorylation at S710 of Kif23-iso2 (S814 in Kif23-iso1) was previously shown to be important for binding to 14–3–3 [25]. We found that both S716A and S814A Kif23-iso1 single mutants were deficient in 14–3–3 binding. While it is still possible that pS716 constitutes a minor 14–3–3 binding site, pS814 is clearly the main 14–3–3 binding site. First, a 26 amino acid including pS814 was found to behave as an autonomous 14–3–3 binder [25], and second, phosphorylation of S716 influences that of S814, but not the opposite. This leads to the conclusion that phosphorylation of S716 indirectly regulates binding to 14–3–3 by influencing S814 phosphorylation, rather than acting as a binding site *per se* for 14–3–3. Such a hierarchical phosphorylation system regulating 14–3–3 binding was reported for the FOXO3/FKHR transcription factor [38]. The underlying mechanism could be that S716 phosphorylation induces unmasking of S814, either directly by changing Kif23 conformation around S814, or by altering binding to an unidentified Kif23 interactor.

Both isoforms of Kif23 reside at the spindle midzone and at the MB, and each is sufficient to achieve proper cytokinesis [25,39]. However, it is possible that each isoform performs other specific non-essential mitotic functions. Besides, Kif23 participates in other cellular processes, where each isoform could carry out different tasks [40,41]. It was reported that the 104 amino acid supplementary domain of Kif23-iso1 bears actin and Arf3 binding activity [42,43]. It could be worth testing if phosphorylation of S716, located inside this domain, and binding to these interactors are interdependent. Interestingly, it was shown that phosphorylation of formin in yeast and angiomotin in human cells by NDR/LATS kinases regulates their binding to actin [21,44]. In any case, phosphorylation of S716, as a requisite for S814 phosphorylation, could modulate binding to 14–3–3 and hence recruitment of Kif23 to the central spindle microtubules. While this regulation could affect only Kif23-iso1, it is tempting to speculate that it could concern both isoforms, as Kif23 was shown to act as clusters [45], let aside the possibility that it could engage as a mixed heterodimer in the centralspindlin complex. Finally, the regulation of Kif23 binding to 14–3–3 by two phospho-sites raises the interesting possibility that this interaction might be controlled by at least two kinases integrating different signaling inputs, as was shown for REEP proteins [46].

At the end of mitosis, the two future daughter cells are connected by the MB, a thin intercellular bridge containing a microtubule rich zone [47]. The MB is organized in sub-compartments, including the central MB ring (also called Flemming body) where Kif23 reside. Asymmetric membrane severing induces the separation of the daughter cells and the inheritance of the MB ring in one of the daughter cells. Alternatively, free floating extracellular MB rings arise from symmetric severing of the cytoplasmic bridge [34,48]. These post mitotic MB rings are referred to as midbody remnants (MB^R^). It was first thought that MB^R^s did not fulfill any specific function and were merely destined to degradation, but recent studies have demonstrated their involvement in post mitotic functions. While in cancer and stem cells, MB^R^s remain in the cytoplasm during multiple cell divisions, in differentiated or differentiating cells, MB^R^s are either degraded by the ubiquitin and autophagosome pathways or expulsed in the culture medium [34,49–51]. Those MB^R^s remaining inside the cell might play a role in cell fate [52]. In differentiating germline stem cells of the drosophila embryo, the MB^R^ is segregated specifically in the daughter cell [53]. In the early C. elegans embryo, the MB^R^ repositions the mitotic spindle in the P1 cell, a crucial step for the dorso-ventral patterning of the embryo [54,55]. In HeLa cells, MB^R^s are rarely released and are mainly found as intracellular organelles with an average life of 11 hrs [49,50,56]. Among the proteins of the MB that persist in the MB^R^ are Cep55, MgcRacGAP and Kif23. In this work, we have shown that Kif23 is largely unphosphorylated on S814 in MB^R^s, in contrast to what is observed for cytokinetic MBs. Since a non insignificant portion of MB^R^s do have relative pS814 levels comparable to those of MBs, it is probable that dephosphorylation of pS814 takes place after abscission, but rather early during the lifespan of the MB^R^s. It will be interesting to determine whether this dephosphorylation occurring in the next cell cycle is the result of lower LATS1,2 kinase activity or higher phosphatase activity, or both. While regulation of LATS1,2 kinases during the cell cycle is still elusive, control of its activity as part of the hippo pathway during control of cell proliferation is well documented. It is worth noting that cell proliferation is correlated with low LATS kinase activity and a high number of MB^R^s. We could show that in the numerous MB^R^s of HeLa cells, phosphorylation of Kif23 on S814, that we suggest to be driven by LATS, is low. On the opposite, we would expect this phosphorylation to be stronger in cells containing fewer MB^R^s. This could merely be the consequence of the expected shorter half-life of MB^R^s in these low-population MB^R^s, the new MB^R^s just arising from abscission being fully phosphorylated on S814. On the other hand, those MB^R^s which escape degradation for longer times would be less phosphorylated on S814, residing in a context of low LATS kinase activity. However, it is tempting to speculate that the phosphorylation state of S814 on Kif23 could itself behave as a determinant of MB^R^s’ longevity. It will be interesting to establish the phosphorylation status of S814 of Kif23 on MB^R^s in other cell types containing different number of MB^R^s and to verify if altering this phosphorylation could modulate the MB^R^s per cell ratio. Alternatively, it could be worth monitoring pS814 to probe MB^R^s during differentiation or embryogenesis.

## Supporting Information

S1 Fig Gel analysiss of NDR1 and LATS2 kinase preparations. GST-NDR1, GFP-LATS2 kinases and kinase-dead (KD) versions were purified from HEK293T cells as described in Materials and Methods and analysed by Coomassie blue staining on polyacrylamide gels. Asterisks denote recombinant GST-GFP-trap and its degradation products.
(TIF)

S2 Fig Phosphorylation of NDR1-MOB1A candidate substrates. 6His-tagged candidate substrate domains, as indicated, were incubated with NDR1-MOB1A kinase (WT or kinase dead (kd)) and analysed by SDS-PAGE and autoradiography.
(TIF)

S3 Fig Characterization of anti-pS716 and-pS814 phosphosite antibodies. Phosphorylated and unphosphorylated peptides lining S716 and S814 were deposited on a nitrocellulose membrane as two-fold serial dilutions and revealed with the corresponding affinity purified ab pS716 and ab pS814.
(TIF)

S4 Fig Kif23 is phosphorylated on S814 on central spindle and midbody ring. Unsynchronised HeLa cells were fixed and stained with ab pS814, anti-Kif23 and anti-tubulin antibodies.
(TIF)

S5 Fig Phosphorylation of Kif23 on S814 is constitutive from S to M phase. HeLa cells were released from a double thymidine block and analyzed for pS814, Kif23 and cyclin B1 content by Western blot. Cyclin B1 was used as a marker of cell cycle progression. A longer exposure (right panel) allowed monitoring of S814 phosphorylation level for the minor isoform 1.
(TIF)

S6 Fig Validation of LATS2 siRNAs on ectopically expressed myc-LATS2. HeLa cells were transfected with myc-LATS2 and control or LATS2 siRNAs (set 1) and analyzed for the amount of myc-LATS2 by Western blot.
(TIF)

S7 Fig Isoforms 1 and 2 of Kif23 have similar S710/S814 phosphorylation levels and 14–3–3 binding properties. Myc-14–3–3 (A, B) and WT or mutant GFP-Kif23-iso1, iso2 (A) were expressed in HEK293T cells and immunoprecipitated with anti-myc antibody. Whole cell extracts as well as immunoprecipitated materials were analyzed by Western blot.
(TIF)

S8 Fig Phosphomimetic S716D mutation does not rescue 14–3–3 binding capacity of Kif23-iso1 S814A mutant. WT and mutant Flag-tagged Kif23-iso1 were expressed with myc-tagged 14–3–3 in HEK293T cells. Material immunoprecipitated with anti-myc antibodies was analyzed by Western blot for the presence of Kif23.
(TIF)

S9 Fig Kif23 and MgcRacGAP co-localise on MB^R^s. Unsynchronized HeLa cells were fixed and stained with anti-Kif23, anti-MgcRacGAP and anti-tubulin antibodies and DAPI. Yellow arrow points to MBs in cytokinetic cells.
(TIF)

S1 Table List of NDR/LATS consensus phosphorylation sites studied. (XLSX)
