# Supplementary figures and images for: Binding of Kif23-iso1/CHO1 to 14-3-3 Is Regulated by Sequential Phosphorylations at Two LATS Kinase Consensus Sites
Source: PLoS One. 2015 Feb 6;10(2):e0117857. doi: 10.1371/journal.pone.0117857 (PMC4320110; doi:10.1371/journal.pone.0117857)

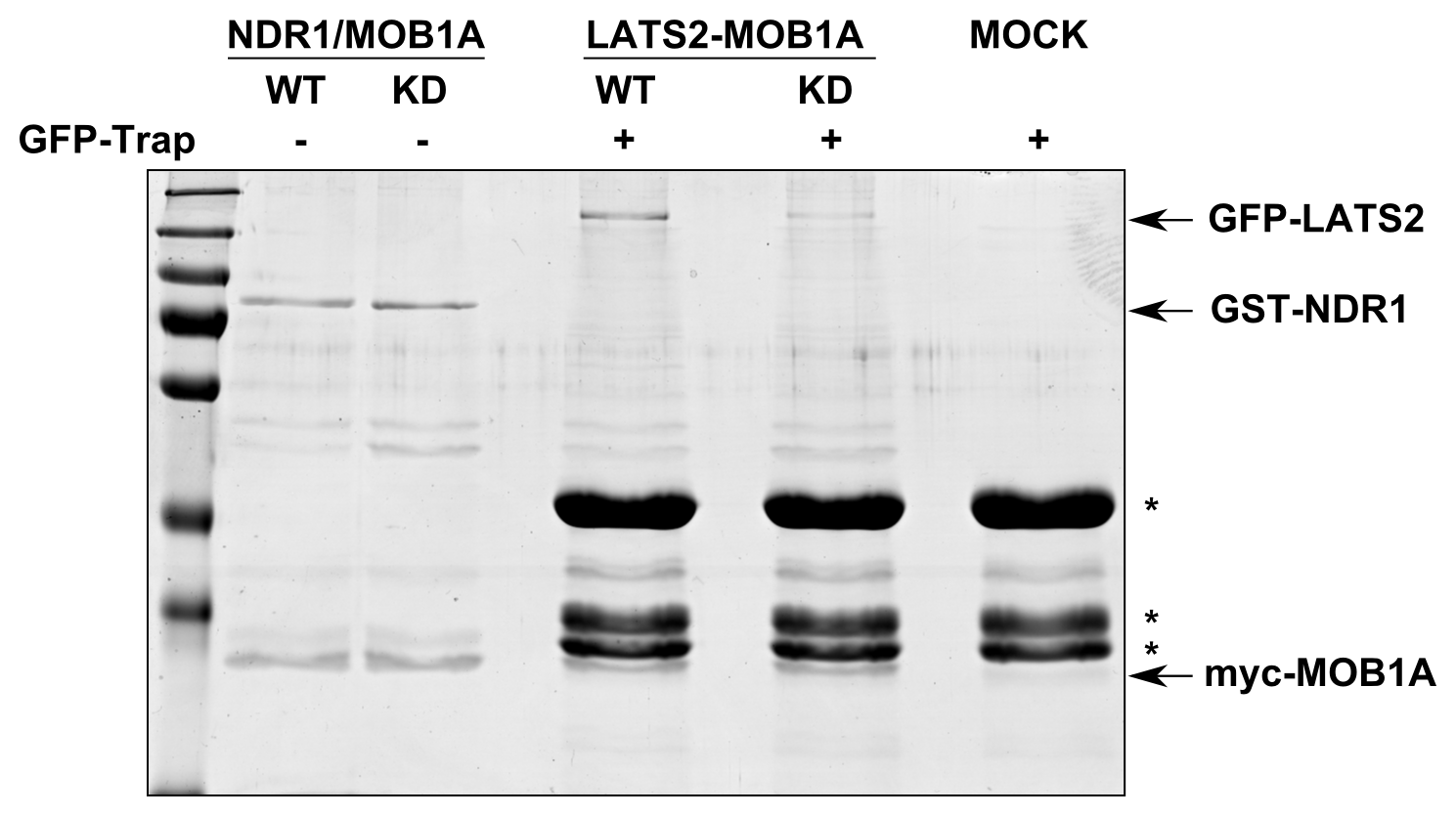

Supplement: S1 Fig — GST-NDR1, GFP-LATS2 kinases and kinase-dead (KD) versions were purified from HEK293T cells as described in Materials and Methods and analysed by Coomassie blue staining on polyacrylamide gels. Asterisks denote recombinant GST-GFP-trap and its degradation products. (TIF) [file pone.0117857.s001.tif]

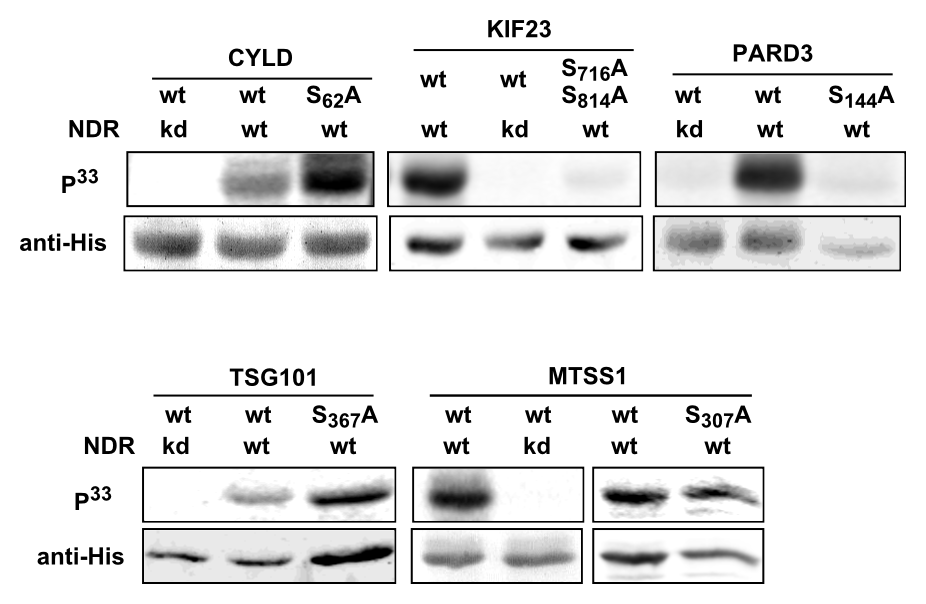

Supplement: S2 Fig — 6His-tagged candidate substrate domains, as indicated, were incubated with NDR1-MOB1A kinase (WT or kinase dead (kd)) and analysed by SDS-PAGE and autoradiography. (TIF) [file pone.0117857.s002.tif]

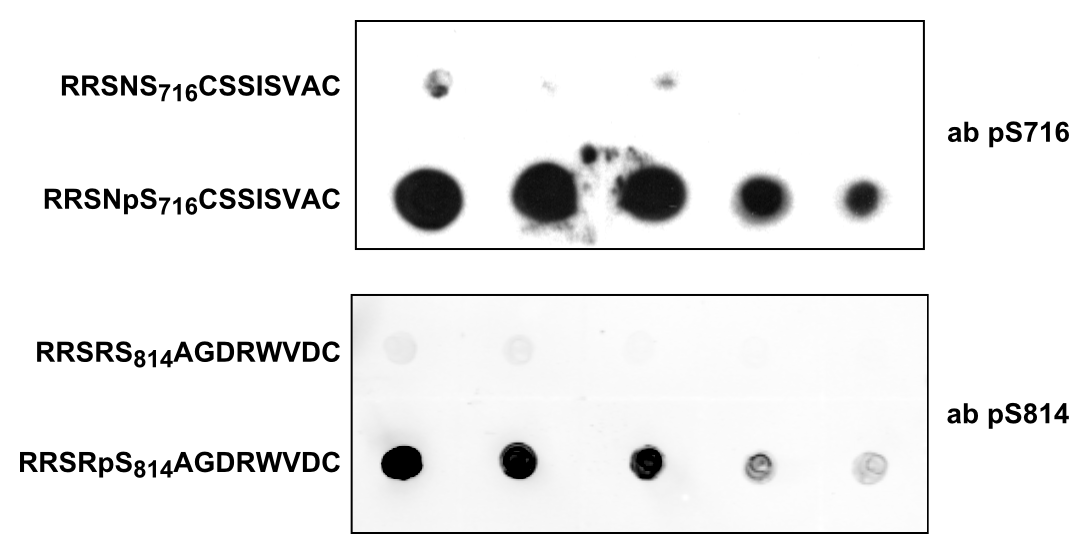

Supplement: S3 Fig — Phosphorylated and unphosphorylated peptides lining S716 and S814 were deposited on a nitrocellulose membrane as two-fold serial dilutions and revealed with the corresponding affinity purified ab pS716 and ab pS814. (TIF) [file pone.0117857.s003.tif]

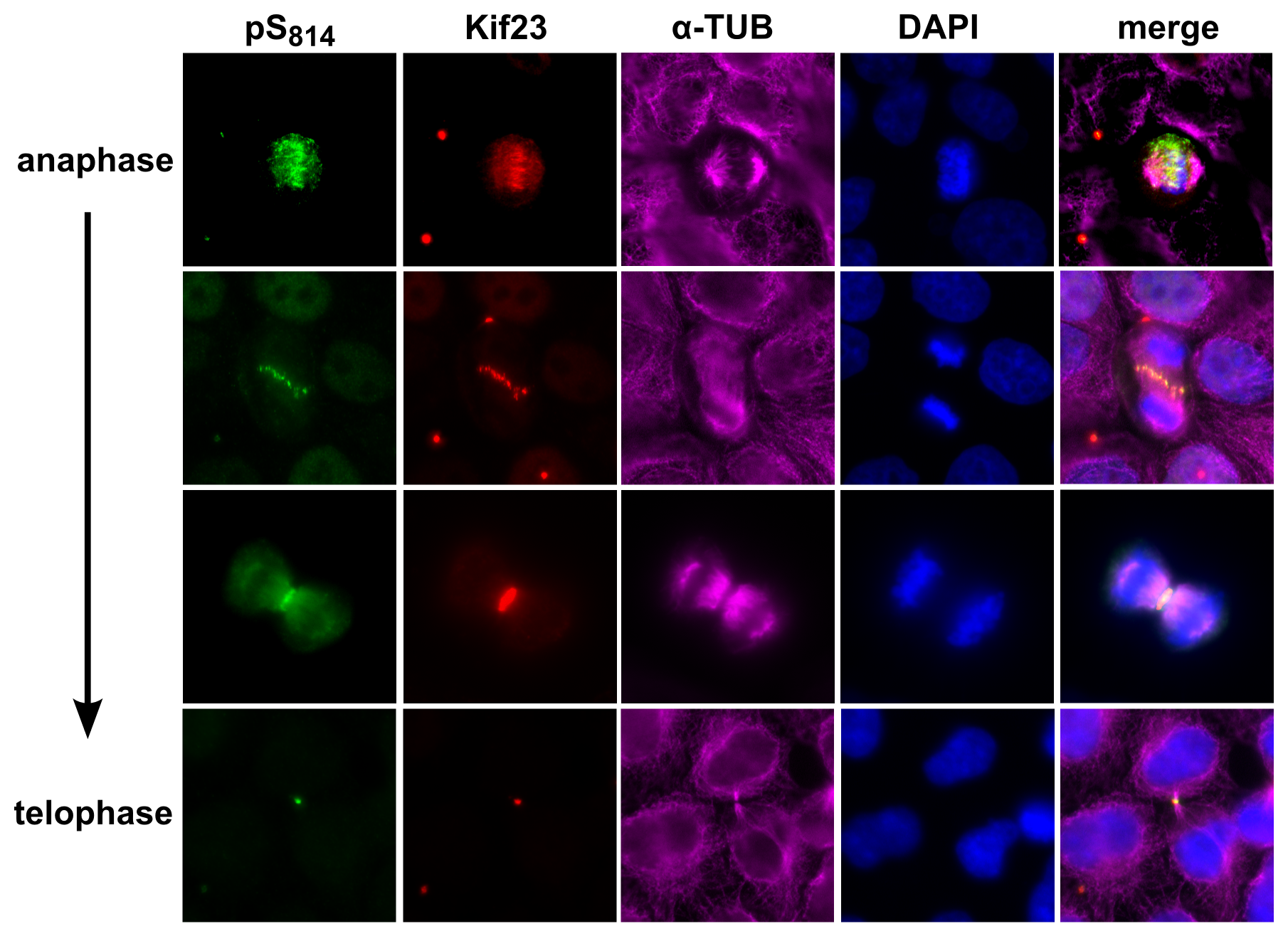

Supplement: S4 Fig — Unsynchronised HeLa cells were fixed and stained with ab pS814, anti-Kif23 and anti-tubulin antibodies. (TIF) [file pone.0117857.s004.tif]

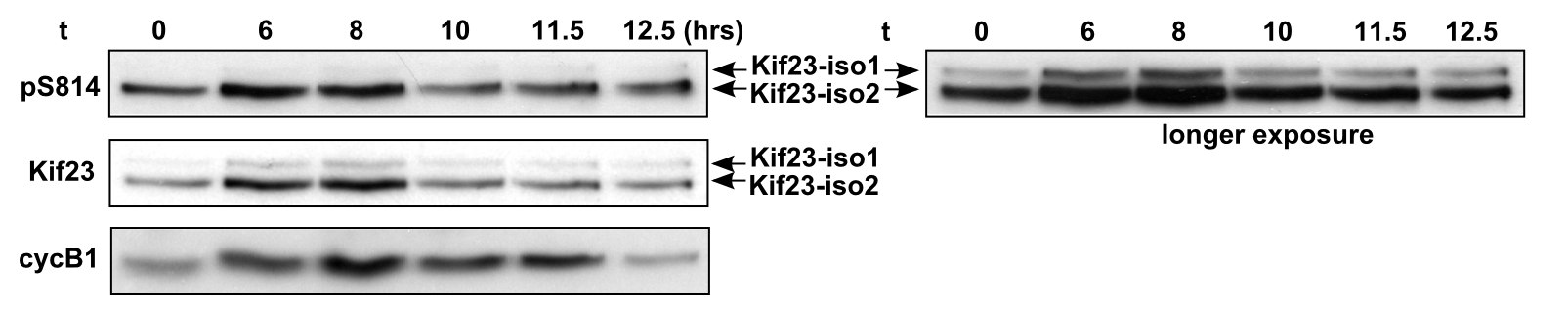

Supplement: S5 Fig — HeLa cells were released from a double thymidine block and analyzed for pS814, Kif23 and cyclin B1 content by Western blot. Cyclin B1 was used as a marker of cell cycle progression. A longer exposure (right panel) allowed monitoring of S814 phosphorylation level for the minor isoform 1. (TIF) [file pone.0117857.s005.tif]

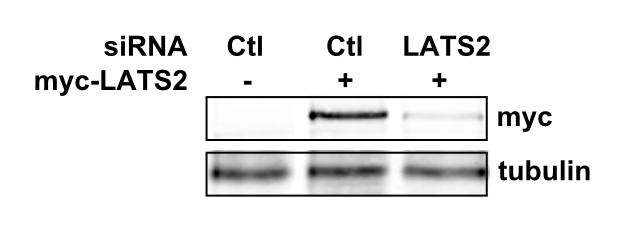

Supplement: S6 Fig — HeLa cells were transfected with myc-LATS2 and control or LATS2 siRNAs (set 1) and analyzed for the amount of myc-LATS2 by Western blot. (TIF) [file pone.0117857.s006.tif]

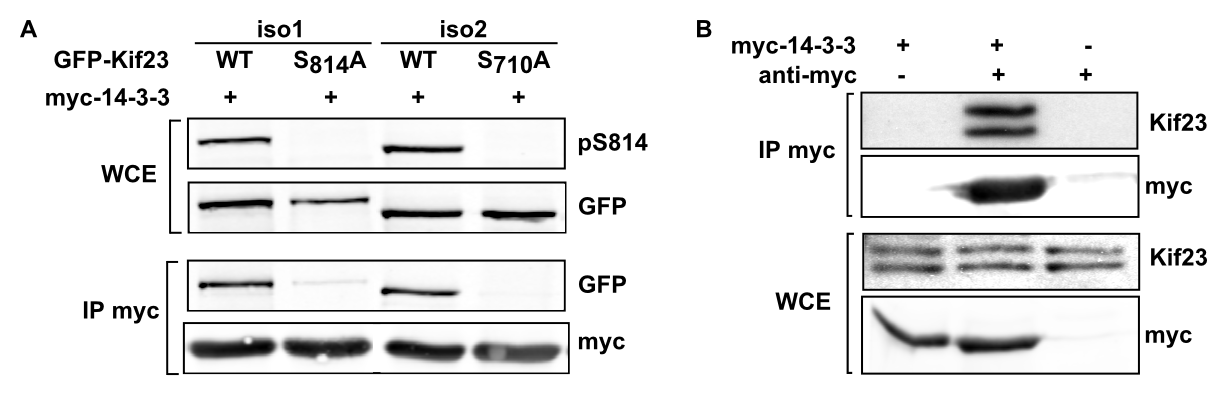

Supplement: S7 Fig — Myc-14–3–3 (A, B) and WT or mutant GFP-Kif23-iso1, iso2 (A) were expressed in HEK293T cells and immunoprecipitated with anti-myc antibody. Whole cell extracts as well as immunoprecipitated materials were analyzed by Western blot. (TIF) [file pone.0117857.s007.tif]

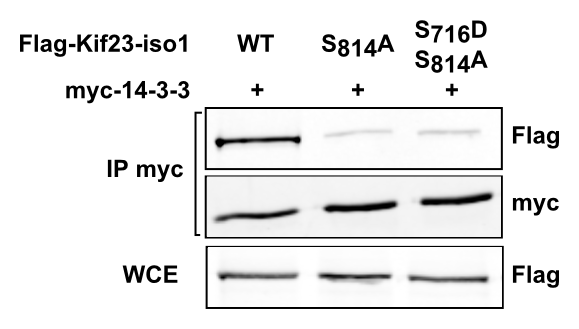

Supplement: S8 Fig — WT and mutant Flag-tagged Kif23-iso1 were expressed with myc-tagged 14–3–3 in HEK293T cells. Material immunoprecipitated with anti-myc antibodies was analyzed by Western blot for the presence of Kif23. (TIF) [file pone.0117857.s008.tif]

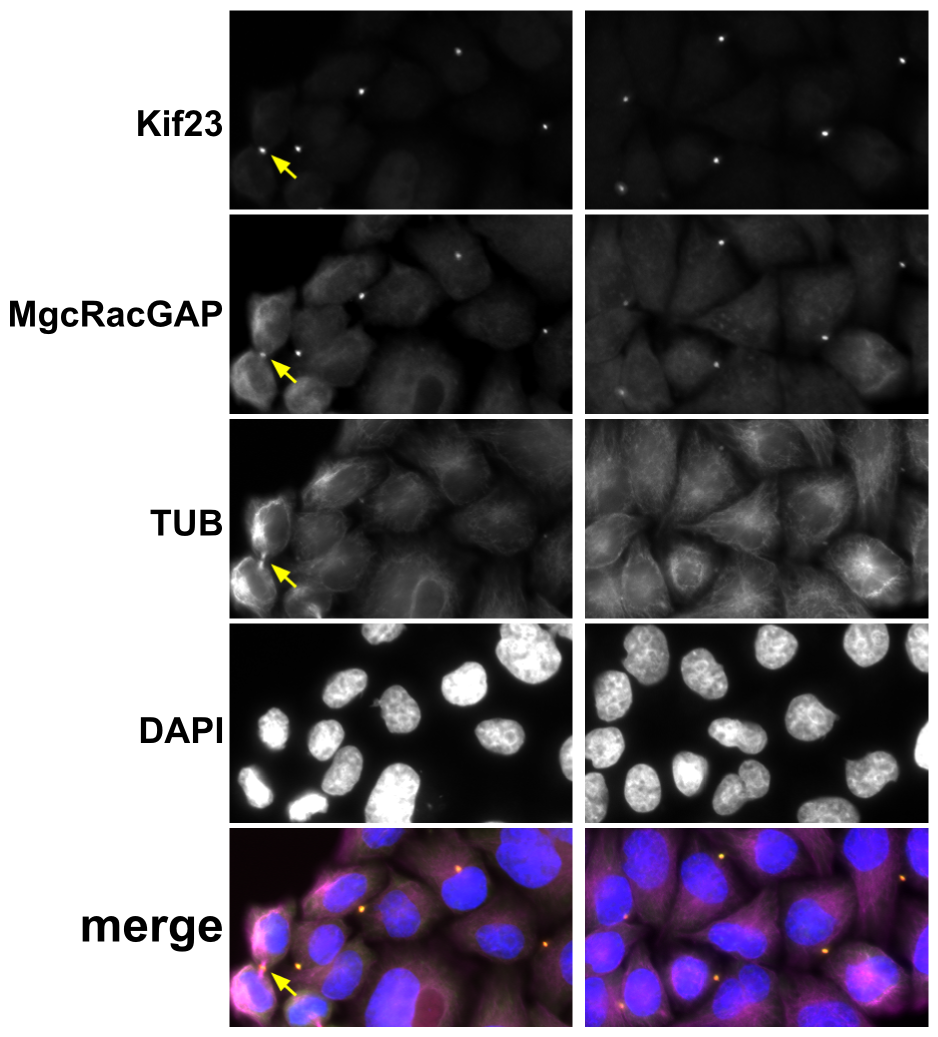

Supplement: S9 Fig — Unsynchronized HeLa cells were fixed and stained with anti-Kif23, anti-MgcRacGAP and anti-tubulin antibodies and DAPI. Yellow arrow points to MBs in cytokinetic cells. (TIF) [file pone.0117857.s009.tif]
